# O069: Efficacy of cleaning methods post-inoculation of pathogenic microorganisms of conventional and novel cleankeys computer keyboards

**DOI:** 10.1186/2047-2994-2-S1-O69

**Published:** 2013-06-20

**Authors:** J De Grood, L Ward, S Harman, C Duchscherer, M Ward, J McClure, K Hope, J Kim, J Vayalumkal, K Zhang, T Louie, J Conly

**Affiliations:** 1 University of Calgary, Canada; 2 Alberta Health Services, Calgary, Canada

## Introduction

Keyboards (KB) may play a role in the spread of healthcare pathogens (HCP). A survey in our hospitals revealed 58.3% (134/230) were + for at least 1 HCP with 60.9% contaminated with fecal organisms.

## Objectives

We sought to determine the adequacy of cleaning methods of conventional and novel acrylic and glass Cleankeys KBs, an innovative, wireless, waterproof KB.

## Methods

Ten conventional and 12 Cleankeys KBs were inoculated with methicillin-resistant *S.aureus* (MRSA),vancomycin-resistant Enterococcus (VRE), *P.aeruginosa* (PA) and *C.difficile* (CD) using a modified technique described by Rutala. Every 2nd key was inoculated with 5x10^3^/50ul of each organism. Cleaning using a standardized protocol was done with CaviWipes™(0.28% quaternary ammonium and 17.2% isopropanol), PCS 1000 Bleach Wipes and Microfibre cloths and dish soap/water. Cultures were obtained with a standardized method using sterile applicators moistened with 0.01M PBS. The applicators were transferred into tubes containing TSB, vortexed, and planted on selective media.

## Results

CaviWipes™ were effective at eliminating MRSA, VRE and PA (97%) from all 3 types of KBs but were ineffective at eliminating CD with 100% of keyboards remaining CD +. PCS 1000 Bleach Wipes and Microfiber cloths eliminated MRSA, VRE and PA from all KB tested, were ineffective for conventional KBs with 100% remaining culture CD + but eliminated CD from both acrylic and glass Cleankeys KBs. Plain dish soap and water were 100% effective at eliminating CD from both acrylic and glass Cleankeys KBs. Conventional keyboards could not be immersed in water.

## Conclusion

KBs represent a high-touch surface for colonization with HCPs. The efficacy of conventional cleaning agents may be suboptimal for conventional KBs harboring CD. An innovative keyboard, washable in plain soap and water offers distinct advantages in promoting hospital hygiene.

## Disclosure of interest

None declared.

